# The Post-Antibiotic Era: A New Dawn for Bacteriophages

**DOI:** 10.3390/biology12050681

**Published:** 2023-05-04

**Authors:** Youshun Jin, Wei Li, Huaiyu Zhang, Xuli Ba, Zhaocai Li, Jizhang Zhou

**Affiliations:** 1 State Key Laboratory for Animal Disease Control and Prevention, College of Veterinary Medicine, Lanzhou University, Lanzhou Veterinary Research Institute, Chinese Academy of Agricultural Sciences, Lanzhou 730000, China; 2 College of Agriculture, Ningxia University, Yinchuan 750021, China; 3 Animal Pathology Laboratory, College of Veterinary Medicine, Northwest A&F University, Xianyang 712100, China; 4 State Key Laboratory of Veterinary Etiological Biology, Lanzhou Veterinary Research Institute, Chinese Academy of Agricultural Sciences, Lanzhou 730046, China

**Keywords:** phage, phage therapy, CRISPR-Cas, antibiotic, synthetic biology

## Abstract

**Simple Summary:**

Phages, also known as bacteriophages, are bacteria-specific viruses that are ushering in a new dawn following the increase in antibiotic resistance. In nature, phages are distributed wherever bacteria exist. They are divided into lytic and lysogenic phages based on their reproduction. Specifically, lysogenic phages reproduce within the bacteria as genetic elements, while lytic phages directly lyse bacteria to release progeny phages. Therefore, lytic phages can be used to treat bacterial infections. However, because the current phage therapy (PT) system has not yet been streamlined, there are still a series of PT-related concerns, such as phage isolation and purification efficiency, the immune response induced by PT, and the impact on intestinal microorganisms. Therefore, synthetic biology, bioinformatics, and artificial intelligence should be combined to edit high-efficiency directionally engineered phages that are safe for humans while effectively killing drug-resistant bacteria.

**Abstract:**

Phages are the most biologically diverse entities in the biosphere, infecting specific bacteria. Lytic phages quickly kill bacteria, while lysogenic phages integrate their genomes into bacteria and reproduce within the bacteria, participating in the evolution of natural populations. Thus, lytic phages are used to treat bacterial infections. However, due to the huge virus invasion, bacteria have also evolved a special immune mechanism (CRISPR-Cas systems, discovered in 1987). Therefore, it is necessary to develop phage cocktails and synthetic biology methods to infect bacteria, especially against multidrug-resistant bacteria infections, which are a major global threat. This review outlines the discovery and classification of phages and the associated achievements in the past century. The main applications of phages, including synthetic biology and PT, are also discussed, in addition to the effects of PT on immunity, intestinal microbes, and potential safety concerns. In the future, combining bioinformatics, synthetic biology, and classic phage research will be the way to deepen our understanding of phages. Overall, whether phages are an important element of the ecosystem or a carrier that mediates synthetic biology, they will greatly promote the progress of human society.

## 1. Introduction

Bacteriophages (phages) were discovered earlier than antibiotics and are the most abundant and diverse organisms on Earth [1,2]. Although phages are one of the simplest biological model systems, their discovery has greatly contributed to basic and applied research in the biological sciences [3]. For example, phages are key contributors to the establishment of the central dogma (DNA → RNA → protein message transfer) [4] and the development of CRISPR-Cas phage resistance systems [5,6].

**Figure 1:**
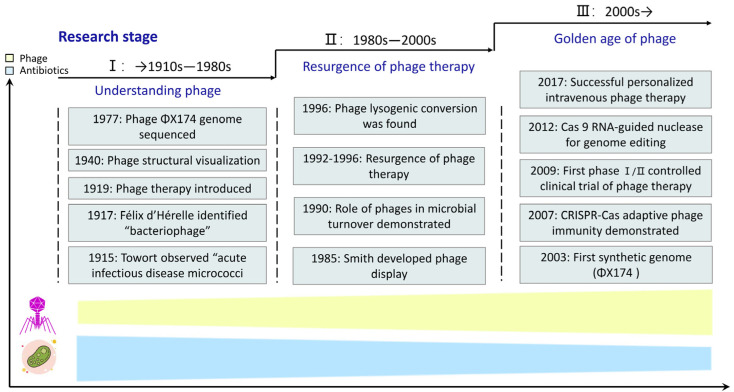
Timeline of some major phage studies.

Moreover, phage therapy was a groundbreaking discovery in medical applications, first used to treat bacterial infections (Figure 1) [7,8]. However, antibiotics gradually replaced phage therapy (PT), phasing out its history and potential. Humans have only been using antibiotics for a century to treat bacterial infections. However, the world faces a terrifying threat of antibiotic resistance [9]. It is estimated that by 2050, 11 to 444 million of the world’s population may die from antibiotic resistance [10,11], resulting in a global GDP and trade loss of 85 and 23 trillion dollars, respectively [12], directly exacerbating global poverty. Furthermore, the extensive use of antibiotics has strengthened horizontal gene transfer through mobile bacterial genetic elements (plasmids, prophages and transposons), inserting a huge selection pressure on the environment (water, soil and atmosphere) [13,14]. For example, there are 200–220 antibiotics in the natural environment [15], including surface water and wastewater species, with an antibiotics concentration of 0.01–1.0 μg [16], which undoubtedly increases the risk of antibiotic-resistant bacteria carrying antibiotic resistance genes [17,18]. Fortunately, research on phage-mediated therapy and synthetic biology continues to deepen. Phages can effectively treat infections caused by antibiotic-resistant bacteria in medicine, agriculture and the food industry. Thus, phages can usher in a new dawn in the post-antibiotic era [7].

Phages are composed of nucleic acid and protein, and eliminate pathogenic bacteria in a targeted manner [19]. However, the efficient lysis of pathogenic bacteria by phages releases lipopolysaccharide, inducing an immune response [20]. In this review, the history of phage discovery, their reproduction cycle and phage clinical applications are discussed in detail. In addition, the effect and safety concerns of PT on intestinal microorganisms and immune responses and the application of synthetic biology on phage gene editing are outlined. This review postulates how phage therapy-mediated synthetic biology, combined with artificial intelligence and deep learning, can be used to treat multidrug-resistant bacterial (MDR) infections precisely.

## 2. Understanding Phages

### 2.1. Phage Discovery

Phages were discovered in the pre-antibiotic era between 1915–1917 by Frederick Twort, a British pathologist, and Félix D’Hérelle, a Canadian microbiologist [21,22]. Subsequently, D’Hérelle isolated Shigella-eaters in 1919, which he named bacteriophages (Figure 1) [22].

Phages are the most widely distributed species in the environment (~10^31^), widely existing in wetlands (~10^9^ g^−1^) and deserts (~10^3^ g^−1^) [23,24]. They are special organisms that cannot reproduce without a host bacteria [25]. Therefore, samples enriched with host bacteria must be selected for bacteriophage isolation. For example, *Pseudomonas aeruginosa* and *Klebsiella pneumoniae* are commonly and rapidly isolated from domestic hospital sewage [26,27]. The speckle method is a traditional phage isolation technique that has greatly advanced the frontier in phage research [28]. Briefly, the host bacteria and phage enrichment solution are cultured in a semisolid medium (0.2~0.5% agar) for 15–20 min and then poured into a solid medium overnight for plaque formation. Although the plaque method has greatly contributed to phage isolation and identification, some phages, such as *Chlamydia*, are difficult to isolate using the plaque method. However, metagenomic sequencing of the viral genome is an effective method for phage identification if the host bacterium has been identified. For example, cross-assembly (CrAss) is an intestinal phage with high host specificity, existing in more than 50% of the bacteria in the human gut. Although it is difficult to isolate the CrAss phage using the traditional plaque method, the putative host bacteria of CrAss and other intestinal bacteria have been identified by metagenomics, which enabled phage isolation [29,30].

Therefore, new sequencing and computing methods may promote the isolation of specific phages [31]. In addition, the phage genome sequences, including their proteins, can be identified or predicted by metagenomics using machine learning or neural network methods, such as Virfinder or MARVEL, based on sequence homologies. Therefore, developing a novel machine learning method supporting stochastic multivariate crossover can accurately predict phage–host interactions and accelerate the isolation of specific phages based on their combined nucleic acid and protein relationships [31].

### 2.2. Phage Classification

Based on their reproductive cycle, phages are classified as either lytic or lysogenic phages [25,32]. Unlike the lytic phages, lysogenic phages integrate their nucleic acid into the host bacteria. Like viruses, the phage genome comprises either DNA or RNA, including double-stranded DNA, single-stranded DNA, double-stranded RNA, or single-stranded RNA [33]. Moreover, phages have few morphological forms, including tailed, tailless, or filamentous, and have a special 20-sided structure. In nature, the tailed phages are the most abundant (~96%) and are further classified as *Siphoviridae* (~61%), *Podoviride* (~14%), and *Myoviridae* (~25%), based on their tail shape and size [33].

### 2.3. Recognition and Infection of Phage

Adsorption, penetration, synthesis (genome replication and protein synthesis), assembly and release are the key phases in phage propagation [34]. Adsorption is the first step in host infection by phages and the key process where the host bacterium resists phage infection. The reversible binding of the phage adhesin to the host fibrous protein induces the baseplate localization, followed by the specific binding of the other tail filament proteins to the host bacteriophage surface receptors, which results in reversible or irreversible binding. For example, the receptor protein gp38 of phage Bp7 reversibly binds Lam B and Omp C proteins of *E. coli* but irreversibly binds the Hep I protein [35]. Moreover, bacteria inhibit phage-specific recognition through genetic mutations that alter their surface receptor structure, number, and other interacting proteins. For example, λ phage alters the protein J terminal structure and binds the new receptor Omp F to complete the subsequent infection [36].

Phage adsorption on the host surface irreversibly injects DNA (or RNA) into the bacteria (host). Subsequently, the phage uses the bacterial material to replicate and assemble in the host bacteria. Blocking the penetration of phage genetic material is the second line of defense against phage infection by the host bacteria. Superinfection exclusion is one of the main mechanisms by which bacteria resist the entry of phage DNA/RNA. The Restriction–Modification Systems and the CRISPR-Cas systems are also key mechanisms by which bacteria resist phage infection. When the phage DNA/RNA first enters the bacterium, exogenous invasive DNA/RNA signature sequences are processed and integrated between two repetitive sequences to form non-repetitive spacer sequences, which are transcribed to form mature CRISPR RNA (crRNA). Subsequently, crRNA directs the Cas protein to cleave the exogenous DNA/RNA precisely, inhibiting phage replication. However, phages also evade the host CRISPR-Cas cleavage by mutating their genetic loci to form anti-CRISPR proteins blocking the Csy complex [37,38]. At the same time, phages such as T4 inhibit other phage reproduction by encoding Imm and Sp proteins, which alters the conformation of the injection site and lysozyme activity [39,40].

## 3. Application of Phages

### 3.1. Gene Editing Using Bacteriophages

Bacterial infectious diseases are detrimental to human health; thus, they are a public health priority globally [41]. In 2019, approximately 13.7 million people worldwide died from infections. More than half of these deaths accounted for 13.6% of the global death, were associated with 33 bacterial infections, including *Staphylococcus aureus*, *Escherichia coli*, *Streptococcus pneumoniae*, *Pseudomonas aeruginosa*, and *Klebsiella pneumoniae*. The death toll from these 33 bacterial infections is second to ischemic heart disease-associated deaths globally [42]. Therefore, preventing mortality following a bacterial infection is key to solving public health problems [41,42]. Although phages can kill bacteria, natural phages have a narrow host range and poor stability. Using synthetic biology, phage isolates can be used to construct engineered microorganisms by editing their genetic information [43,44], such as altering their host range, transforming between lysogenic and lytic phages, modifying lysozymes, and increasing phage stability.

**Figure 2:**
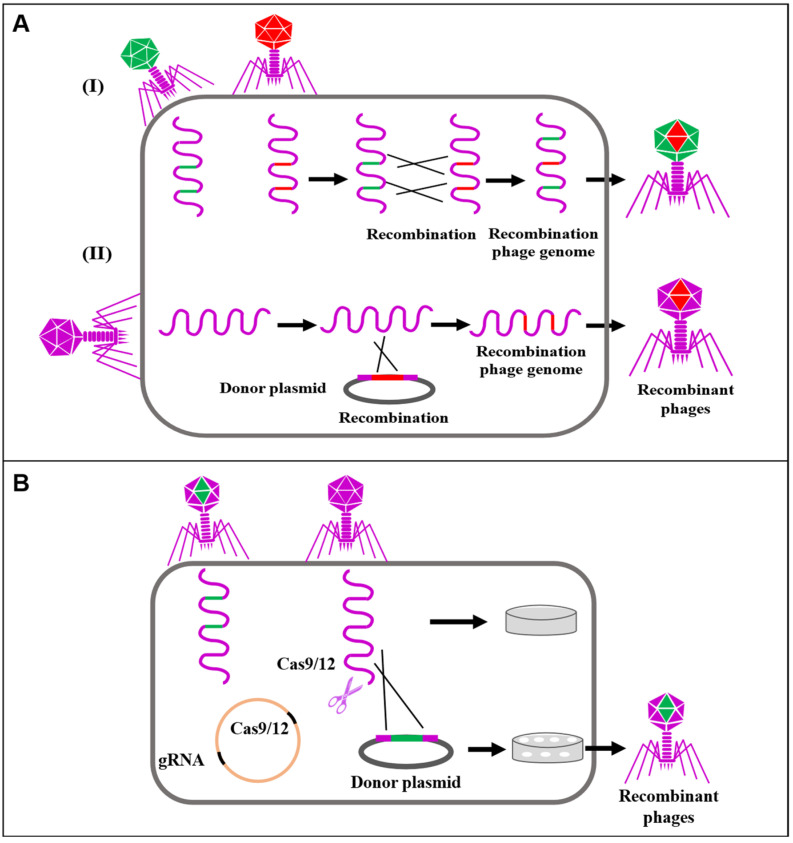
Application of synthetic biology in phage gene editing: (**A**) Transformation of phages through homologous recombination (**I**) Genetic recombination between plasmids without homologous sequences and genes of invading bacteriophages, (**II**) Genetic recombination between plasmids containing homologous sequences and phages invading the bacteria. (**B**) Editing phages using CRISPR-Cas. Red, green and purple represent the phage species carrying different genes.

Whole-genome sequencing is essential to identifying engineered phages. Homologous recombination is a natural phenomenon widely existing in the biological world. When two sequences have homologous fragments, foreign genes can be integrated into the target genome through homologous recombination [45,46]. During homologous recombination, the exogenous target gene is cloned into a plasmid. Next, a plasmid–phage hybrid is constructed where the phage carrying the donor plasmid infects the host bacteria to complete the homologous recombination (Figure 2). The principle of homologous recombination lies in the collision of homologous genes. However, the homologous recombination occurs only in some progeny phages (10^−10^~10^−4^), requiring labor-intensive screening of target phages [45]. As a result, the luciferase, fluorescent protein gene, or resistance gene is introduced into the recombinant phage during recombination to recognize mutant phages [47] specifically. Since their discovery more than a decade ago, the CRISPR-Cas systems have revolutionized patterning studies in biological research [47]. The CRISPR-Cas system consists of three major steps: adaptation, crRNAs biosynthesis, and interference. The CRISPR-Cas system is a prokaryotic immune system which uses crRNAs and Cas nucleases to recognize and destroy foreign nucleic acids [48]. Briefly, foreign nucleotide sequences (30~40 nucleotide) called “spacers” are captured and integrated into the CRISPR loci between palindromic DNA repeats. Subsequently, the spacers are transcribed into precursor crRNAs and further processed to release mature crRNAs. Finally, the crRNAs bind the Cas proteins, which specifically recognize and degrade the complementary crRNAs, constituting the immune defense of CRISPR-Cas [5,48]. Currently, several CRISPR systems (I and II) transform phages through gene editing to infect diverse hosts [49,50]. The CRISPR-Cas systems also induce counter-selection on phages, which enables structural recombination with the donor DNA through transposition [50,51]. The donor DNA contains a segment of the phage genome and the target gene, with homologous sequences of the phage genome on both sides. Thus, the engineered phage can escape the immunity of the CRISPR-Cas systems and complete its life cycle [52]. It is worth noting that although this method greatly enriches rare engineered phages, the recombination rate is still a major limiting factor (Figure 2).

### 3.2. Phage Therapy

PT is booming in many countries worldwide, including Poland, China and Belgium. The development of PT was tortuous in the early days, mainly because antibiotics were used to treat bacterial infections shortly after the discovery of phages [3]. Although the discovery of antibiotics has largely guaranteed the safety of human life, the discovery of drug-resistant bacteria calls for the development of alternative antibody products similar to PT [53,54]. Among the advantages of PT is that phages are easy to separate and purify. Once used for treatment, phages automatically replicate, increasing their number; thus, they are a “live drug”. More importantly, phages do not attack any other cells, which largely guarantees the safety of PT [19,55].

**Figure 3:**
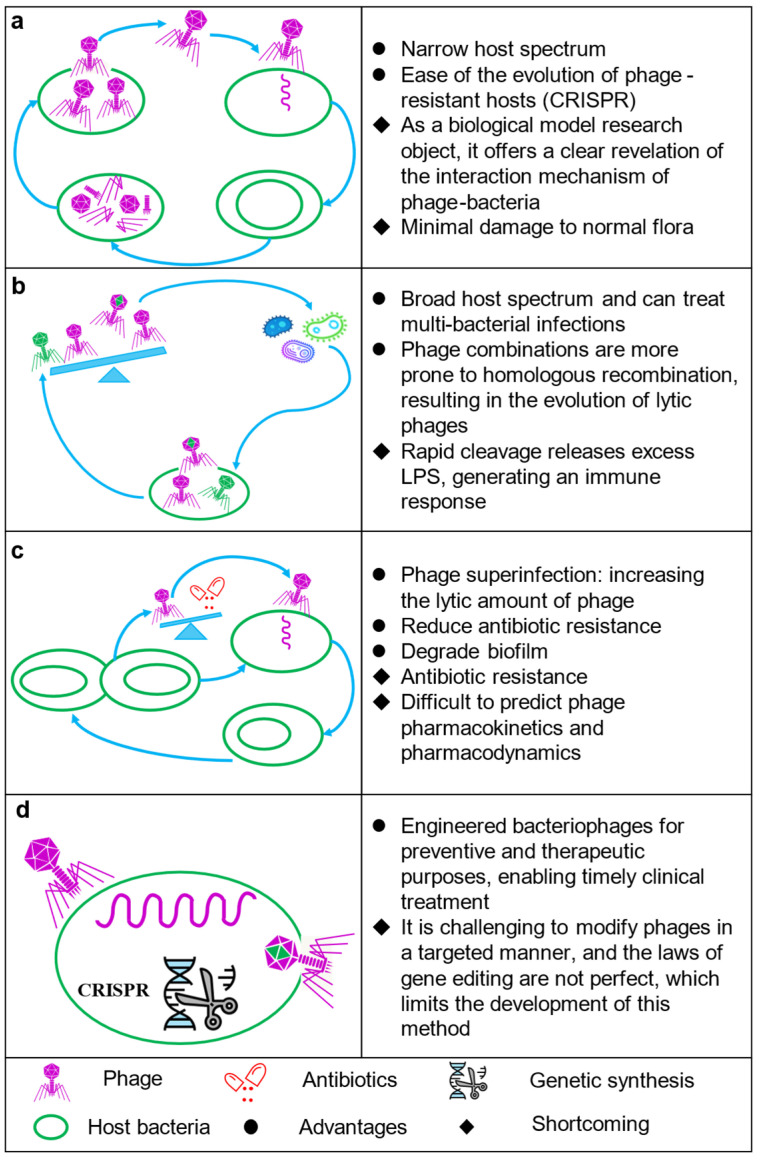
Clinical applications of PT (**a**) Individual PT, (**b**) Phage cocktail therapy against bacterial infection, (**c**) Combination of PT and antibiotics for treating bacterial infection, (**d**) Synthetically engineered phages for treating drug-resistant bacterial infections.

PT includes individual phage therapy (IPT) and multi-phage combination therapy (MPT). IPT is mainly used to understand the lysis mechanism of MDR bacteria in the laboratory. Undeniably, a co-evolutionary relationship exists between phages and host bacteria, which could lead to phage-resistant bacteria and affect IPT applications. Therefore, PT can be administered as MPT (phage cocktails), combining phages and antibiotics, or engineered phages (Figure 3). At present, the FDA has approved some new phage preparations developed by Adaptive Phage Therapeutics (https://www.aphage.com/science/pipeline/ accessed on 16 April 2023) and Intralytix (http://intralytix.com/index.php?page=hum accessed on 16 April 2023) [56,57]. These phage preparations have many common advantages, such as treating acute and antibiotic-resistant bacterial infections, improving the quality of life, and balancing intestinal flora, which significantly improves the clinical efficiency of PT [58]. However, given the host specificity of phages, they also have some unavoidable disadvantages. For example, Intralytix mainly targets adherent invasive *E. coli* associated with Crohn’s disease, *Shigellosis*, and antibiotic-resistant enterococci *Bacteremia*, while Adaptive Phage Therapeutics is best at treating infections caused by *Acinetobacter baumannii* and *Pseudomonas aeruginosa* [58,59].

#### 3.2.1. Phage Immune Mechanisms

PT causes bacterial death by rupturing. However, the rapid bacterial lysis facilitates the diffusion of endotoxins or inflammatory factors into the host body, inducing immune responses such as stimulating Toll receptors (such as TLR 3 and 9 signaling) [53,60,61,62]. Precisely, the phage-induced inflammatory factors are influenced by the phage species, degree of purification, type of combination, and method of administration [63,64]. Innate immunity, the first line of immune defense in mammals, recognizes microorganisms through recognition receptors on the cell surface [53]. Unlike bacterial glycoproteins and polysaccharides [65], bacteriophages have an extremely simple biochemical composition (proteins and nucleic acids) [53]; thus, they do not fully stimulate the pattern recognition receptors. However, some phages are also phagocytized by the blood and heart or cleared by dendritic cells in vitro [66]. For example, adding phage solution to the diet reduces interleukin (IL)-1β and tumor necrosis factor-α in weaned piglets but does not affect interferon-γ [67]. In addition, the oral administration of phages increases the CD^4+^ and CD^8+^ cells producing cytokines. It also induces the production of pro-inflammatory factors IL-12 and IL-6 and anti-inflammatory factor IL-10 by dendritic cells in vitro [61]. The lipopolysaccharide produced during phage lysis of host bacteria (usually less than the lipopolysaccharide released following antibiotic-based therapy) also induces the pro-inflammatory effect [68,69], with excessive lipopolysaccharide release inhibiting the therapeutic effect of phages [70]. Therefore, the interaction between phages and host-specific immune and epithelial cells results in the feedback regulation of phages [55,71]. For example, phages are recognized by the RIG-I receptor of antigen-presenting cells, increasing the IL-15 through the MAVS-IRF-1 signaling pathway, which enhances the CD8ααα + TCR-αβ+ and CD8αβ + TCR-αβ+ epithelial lymphocytes activity and function [71]. Moreover, phage-specific IgG or IgA, including high Ab levels and Fc receptor-mediated uptake of phage/Ab complexes by macrophages, knock out some phages, limiting phage proliferation [53,66,72].

#### 3.2.2. Phages in the Gut

The gastrointestinal tract is the body most densely populated with microorganisms, with a phage-to-bacteria ratio of 10:1 [73,74]. However, this ratio is closer to 1:1 in the intestinal tract (average 10^8^~10^9^ virus-like particles and ~10^9^ bacteria per gram of feces) [75], which is lower than in the ocean [73,76]. Thus, phages mostly exist as lytic phages in the ocean and lysogenic phages (contributing 20% of the host genome) in the gut [74]. Lysogeny is called the phage refuge [64], effectively integrating the host bacteria DNA/RNA while avoiding capture by CRISPR-Cas elements [76]. Moreover, lysogenic phages are more likely intestinal regulators whose release is induced by ultraviolet light, antibiotics, and short-chain fatty acids, broadening their ecological niche in the gastrointestinal tract [64,74]. Regardless of their number, the free phages increase their predation pressure on the intestinal host bacterial community, altering the phage–bacteria relative abundance [77].

In the intestinal microecological environment, mining phage–bacteria interactions may better reveal the role of phages in shaping the intestinal bacterial community and human health [74,76]. The phage characteristics, including their number, diversity, host range and stability, undoubtedly restrict the understanding of the microbiome [74,78]. Nevertheless, we can still adopt the concept of ecology to understand the existence of phages in the gastrointestinal tract. For example, phages play the role of predators. As in macroecology, phages also have a parasitic or mutualistic relationship (lysogenic phages), maintaining a dynamic balance for the age, health, and living environment of people or animals. The global overuse of antibiotics is causing serious food safety problems, which means we may be ingesting low doses of antibiotics inducing lysogenic phages to release them. For example, the phages in the dung of an antibiotic resistant (ciprofloxacin and ampicillin) mouse model encoded *NORM*, *mexD*, and *mexF* genes, implying that the phages mediated antibiotics and regulated the drug resistance in the drug-resistant bacteria [79,80]. The phages also mediated the gene transfer by lysing bacteria and reshaping the ecological structure [55,80,81].

Phages usually only recognize specific bacteria [34]. Contrary to the initial hypothesis of lytic phages inducing intestinal dysbiosis by killing bacteria, the enteroaggregative *E. coli* (EAEC) reduced the β-diversity of microorganisms. However, EAEC mortality induced by *Myoviridae* phage PDX did not lead to microbial dysbiosis, suggesting that phage–bacteria interactions are nested and modular in specific environments such as the gastrointestinal tract [82]. In addition, phages regulate the negative effect of reduced host bacteria through a cascade reaction [55,83]. Interestingly, phages also act as special “workers” in the body. Research has shown that phages may be involved in the synthesis and degradation of cell walls and the gene encoding of anaerobic nucleotides [73]. Moreover, several phages are involved in synthesizing and transporting carbohydrates [69], which has epoch-making significance in studying microbial material and energy cycles [73,76].

#### 3.2.3. Biosafety of Phage Therapy

Unlike other drugs such as antibiotics and lysozyme, phages may have special safety risks as viral therapeutic drugs [84,85]. Since phages encode virulent genes, they may act as carriers of harmful genes through lysogeny transduction, leading to new drug-resistant bacteria. Phages also promote inflammation and trigger immune responses. Moreover, as a therapeutic drug, PT administration (oral, dose, form and site of action) may also lead to a series of potential risks [86,87,88]. For example, repeated administration of phages could promote the evolution of host bacteria resistant to phages [85]. More importantly, whether PT accurately delivers phages to the target site should be considered [89]. This is with respect to the oral administration of phages, which could impact their efficiency in penetrating cell barriers. PT to treat persistent infections caused by MDR bacteria can also produce biofilms, such as cystic fibrosis caused by binding mycobacteria [90]. Therefore, phage packaging needs further considerations, including safe phage delivery systems such as nanoliposomes, microemulsions and hydrogels [89,91,92]. Overall, the safety and efficacy of PT must be evaluated in many ways to establish a safe PT system (Table 1).

## 4. Conclusions and Future Directions

Since their discovery, phages have greatly advanced the human understanding of the microscopic world. Phages are not only used as a model for biological research but also as a vehicle for world-changing hand-synthetic biology. Although numerous research on phages has been conducted, very little is known about them, and a lot is yet to be established, including the significance of phages in the ecosystem, why lytic and lysogenic phages exist, and the prerequisites for their switch. In addition, the protocols to prepare high-efficiency phage cocktails against clinical drug-resistant bacteria are yet to be developed. In the future, the development of PT may focus on the following areas:

- Precise treatment: Synthetic omics can use phages as biological agents to treat MDR bacterial infections and antigen delivery to induce specific immune responses in the body.
- Regulating gut microbes: Gut phages can influence the host response by altering the composition of gut biota. However, theoretical models are still needed to support the phage-induced disturbance responses, including the mechanism of repairing and maintaining gut biota stability.
- Antibiotic alternatives: Phages can be used as antibiotic substitutes or supplements in animal husbandry, including daily healthcare and slaughter of livestock and poultry, to prevent food-borne bacteria from entering the food chain.

Future studies should combine artificial intelligence, deep learning, multi-omics integration and correlation, synthetic biology and other interdisciplinary studies to establish the phage–bacteria interaction and regulation mechanism, develop precise gene editing methods, build a phage reverse genetics platform, establish directed editing of high-efficiency phages that meet human needs and, combined with biological information, design phage cocktails for clinical applications. Phages are not only a model object for biological research but could also usher in a new dawn in medicine, ecology, agronomy and pharmaceutics.

## Figures and Tables

**Table 1:** Summary of clinical trial results for PT.

Year	Country	Clinical Condition and Number of Patients Treated with Phages	Targeted Bacterial	Phages Cocktail (Yes or No)	Results	References
2022	United States of America	Two or more positive mycobacterial cultures in at least one organ (based on ATS/ERS/ESCMID) (*n* = 20)	Non-tuberculous Mycobacterium	Yes (*n* = 9)	11 patients were assessed as responding well, five patients could not be assessed for treatment effect, and four patients showed no significant improvement	[93]
2022	France	Discitis with spinal abscess (*n* = 1)	Multidrug resistant *Pseudomonas aeruginosa*	Yes (*n* = 3)	The patient could walk without pain and has a good prognosis	[94]
2018	Georgia	The patient suffered from respiratory complications, including intermittent infections caused by *Pseudomonas aeruginosa* and *Staphylococcus aureus* (*n* = 1)	*A. xylosoxidans*	Yes (*n* = 2)	The patient’s self-consciousness significantly improved, dyspnea disappeared, and cough was relieved	[95]
2022	United States of America	Treatment of chronic sinusitis and recurrent ear infections in a woman with a history of diabetes and sarcoidosis (*n* = 1)	Methicillin-resistant *S. aureus*	No	The symptoms were alleviated without signs of relapsing chronic sinusitis or otomastoiditis	[96]
2021	India	Severe pain in the right testicle, radiating to the right buttock, right lower back, and left and right pelvic area. Perineal pain, accompanied by sweating, general weakness and physical discomfort (*n* = 1)	*S. aureus* and *S. mitis*	Yes	The patient is in full remission	[97]
2021	China	The patient had a long history of type 2 diabetes and had recurrent lung infections during the past two years of hospitalization due to repeated use of mechanical ventilation (*n* = 1)	Carbapenem-resistant *A. baumannii* (CRAB)	No	No re-emergence of CRAB was observed, and the patient remained stable	[98]

## Data Availability

Where no new data were created.
